# A phase I, dose-escalation study of TB-403, a monoclonal antibody directed against PlGF, in patients with advanced solid tumours

**DOI:** 10.1038/bjc.2011.609

**Published:** 2012-01-24

**Authors:** U Lassen, D L Nielsen, M Sørensen, L Winstedt, T Niskanen, Y Stenberg, S Pakola, J-M Stassen, S Glazer

**Affiliations:** 1 Department of Oncology 5073, Rigshospitalet, Blegdamsvej 9, 2100 Copenhagen, Denmark; 2 Department of Oncology, Herlev Hospital, Herlev Ringvej 75, 2730 Herlev, Denmark; 3 BioInvent International AB, Solvegatan 41, 22370 Lund, Sweden; 4 ThromboGenics, Gaston Geenslan 1, 3001 Heverlee, Belgium

**Keywords:** antiangiogenesis, dose escalation, pharmacokinetics, tolerability, TB-403, RO 5323441

## Abstract

**Background:**

TB-403 (RO 5323441), a humanised monoclonal antibody, is a novel antiangiogenesis agent directed against placental growth factor. The safety, pharmacokinetics (PK), and antitumour activity of TB-403 were assessed in a phase I, dose-escalation study in patients with advanced solid tumours.

**Methods:**

Patients in sequential dose groups received either weekly doses of 1.25, 5.0, or 10 mg kg^−1^ or doses of 20 or 30 mg kg^−1^ every third week.

**Results:**

Twenty-three patients were enrolled and received TB-403. The most common adverse events (AEs) were fatigue, constipation, pyrexia, dyspnoea, and nausea. One serious AE, a lung embolus in a patient with non-small cell lung cancer treated with 10 mg kg^−1^ weekly, was deemed possibly related to TB-403. No dose-limiting toxicities were observed, and a maximum-tolerated dose was not reached. The PK parameters were dose linear and the terminal half-life values ranged from 9 to 14 days. Six patients exhibited stable disease for at least 8 weeks. Two patients, (oesophageal squamous cell carcinoma and pancreatic adenocarcinoma) both treated with 5 mg kg^−1^ weekly, remained stable for 12 months.

**Conclusion:**

TB-403 treatment in this patient population is well tolerated, with a safety profile distinct from that of vascular endothelial growth factor-axis inhibitors.

Angiogenesis (new blood vessel formation) has a critical role in the development and maintenance of cancer and it is widely recognised that tumours must induce angiogenesis to grow beyond a size of 1–2 mm in diameter, as well as to metastasise to distant organ sites (Carmeliet, 2003; Ferrara and Kerbel, 2005). Inhibition of angiogenesis as a therapeutic strategy in oncology has been validated by treatments that block vascular endothelial growth factor (VEGF) or its receptors and several antiangiogenesis inhibitors have been approved (Hurwitz *et al*, 2004; Demetri *et al*, 2006; Escudier *et al*, 2007). Angiogenesis inhibitors have been shown to prolong progression-free survival and/or survival in various cancer patients when combined with chemotherapy. However, during treatment with VEGF(R) inhibitors the tumours may develop resistance to such therapies and others are insensitive to VEGF blockade, suggesting that inhibition of additional targets may be necessary to achieve better clinical effects (Kerbel, 2008).

Placental growth factor (PlGF) is a pleiotropic cytokine that stimulates endothelial cell growth, migration, and survival; chemoattracts angiocompetent macrophages and bone marrow progenitors; and determines the metastatic niche (Fischer *et al*, 2007). It is specifically upregulated in pathological angiogenesis, such as that in cancer but also in eye diseases caused by neovascularisation, and chronic inflammatory conditions (Luttun *et al*, 2002; Autiero *et al*, 2003). Placental growth factor levels in plasma and tumours correlate with tumour stage, vascularity, recurrence, metastasis, and survival in various tumour diseases (Ho *et al*, 2007; Fischer *et al*, 2008; Pompeo *et al*, 2009; Escudero-Esparza *et al*, 2009, 2010; Nagaoka *et al*, 2010). It is also upregulated in human tumours after radio-immunotherapy (Taylor *et al*, 2003), as well as during treatment with VEGF(R) inhibitors, suggesting a mechanism of resistance (Willett *et al*, 2005, 2009; Deprimo *et al*, 2007; Rini *et al*, 2008; Dror Michaelson *et al*, 2009; Kopetz *et al*, 2010; Gerstner *et al*, 2011).

It binds to VEGF receptor 1 (VEGFR-1) and inhibition of PlGF or VEGFR-1 has been reported to reduce tumour growth and angiogenesis (Carmeliet *et al*, 2001; Hiratsuka *et al*, 2002; Fischer *et al*, 2007). Genetic studies in mice show that PlGF is redundant for vascular development and maintenance, but contributes to the angiogenic switch in disease (Carmeliet *et al*, 2001). Studies with an antimouse PlGF antibody showed no effect on fetal development, hypertension, regression of healthy vessels, or increased risk of thrombosis in mice; thus, it is assumed that targeting PlGF will be devoid of the side effects associated with VEGF(R) inhibitors (Eskens and Verweij, 2006; Fischer *et al*, 2007). Safety of antiangiogenic treatments is of key importance, especially as they are used in combination with other anticancer agents.

TB-403 is a humanised recombinant IgG_1_ monoclonal antibody expressed in Chinese hamster ovary cells and directed to the receptor-binding site of PlGF. The antibody was selected based on its high affinity binding to PlGF and significant inhibition of PlGF binding to VEGFR-1, *in vitro*. TB-403 has been shown to significantly inhibit tumour growth in xenograft tumour models and is thought to act in a pleiotropic manner and with a complementary mechanism to VEGF(R) inhibitors (Fischer *et al*, 2007; Rizzo *et al*, 2010). The pharmacokinetic (PK) and toxicological properties of TB-403 have been studied in relevant, non-clinical systems creating the basis for a safe introduction of the compound to clinical studies.

In a previous, first-in-human study, healthy male subjects were given single doses of TB-403 or placebo at the dose levels: 0.3, 1.25, and 5 mg kg^−1^ body weight (BW). No serious adverse events (SAEs) or adverse events (AEs) related to treatment were reported (Martinsson-Niskanen *et al*, 2011). Here we report the results of a Phase I study investigating the effects of multiple doses of TB-403 for the first time in cancer patients. The study assessed the safety, tolerability and pharmacokinetics of intravenous TB-403 in patients with advanced solid tumours.

## Materials and methods

### Study design

This was a Phase I, open-label, dose-escalation, multicentre study in patients with solid tumours. Patients were divided into five different dose groups each comprising three patients. In order to obtain sufficient PK/PD data at the highest dose and to direct further development in malignant diseases, the highest dose group was expanded to nine patients. Patients received 100 ml TB-403 as an intravenous infusion, using an infusion pump, over one hour. Each patient underwent evaluation of ECG, vital signs and temperature before treatment, during infusion, and 4 h after each infusion. After the first infusion, patients were hospitalised for 24 h, during which serial PK samples were taken.

**Figure 1:**
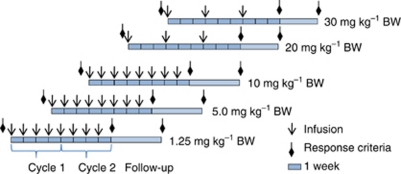
TB-403 was given as eight weekly infusions in the first three dose groups: In dose groups 5 and 5, three doses of TB-403 were given with an interval of 3 weeks. Objective tumour response was evaluated by physical examination and CT on week 8 and week 12 according to RECIST 1.0. Patients with objective response/stable disease were offered extended therapy until disease progression with CT scans every sixth week.

Patients in the first three dose groups were dosed weekly with eight doses of 1.25, 5.0 and 10 mg kg^−1^ BW, respectively (Figure 1). Based on the evaluation of PK data from the first dose groups, the protocol was amended and the dose regimen was changed so that patients in dose groups 4 (20 mg kg^−1^ BW) and 5 (30 mg kg^−1^ BW) received three infusions with an interval of 3 weeks (Figure 1). The longer dosing interval introduces a greater flexibility with either weekly or q3w dosing, allowing TB-403 to be combined with other anticancer agents in future studies. Objective tumour response was evaluated by physical examination and CT at screening and on week 8 and week 12 according to RECIST 1.0. Patients with objective response/stable disease were offered extended therapy until disease progression with CT scans every sixth week.

### Ethics

The study was approved by The Regional Ethics Committee, and all patients have given their written, informed consent; ClinicalTrials.gov Identifier: NCT00702494.

### Eligibility criteria

Inclusion criteria included histologically confirmed malignancy (measurable disease, solid tumour) that was metastatic or unresectable and for which standard curative or palliative treatment did not exist or were no longer effective; ECOG performance status ⩽1; life expectancy ⩾3 months; adequate haematologic function (WBC ⩾3000 per mm^3^, absolute neutrophil count ⩾1500 per mm^3^, haemoglobin ⩾6.0 mmol l^−1^, and platelet count ⩾100 000 per mm^3^), hepatic function (bilirubin ⩽1.5 times upper limit of normal (ULN), AST and ALT ⩽2.5 times ULN), and renal function (normal creatinine clearance related to age and body surface; determined by CrEDTA or Cockcroft formula).

Exclusion criteria included concurrent secondary malignancy; severe or uncontrolled renal condition, cardiovascular disease (NYHA Class ⩾III); unstable angina, congestive heart failure, cardiac arrhythmia; uncontrolled hypertension; and antitumour treatment within 4 weeks before inclusion.

### Evaluation of safety

All patients were seen weekly for 12 weeks (treatment period and follow-up) and underwent physical examinations including ECOG performance status, vital signs, and laboratory examination (including blood count, clinical chemistry, pro-thrombin time/partial thromboplastin time and urinalysis). Toxicities and AEs were monitored using the CTCAE version 3.0 (www.ctep.info.nih.gov/reporting/ctc.html). Serum was analysed on day 130 to check for any late appearing anti-TB-403 antibodies. Patients on extended treatment (after week 8, dose groups 1, 2, and 3, or after week 9, dose groups 4–5) were evaluated for safety before each dosing.

### Definition of dose-limiting toxicity and maximum-tolerated dose

Dose-limiting toxicity (DLT) was defined during the first 4 weeks as one of the following events, provided the toxicity was at least possibly associated with TB-403: grade 4 neutropenia; grade 4 thrombocytopenia; non-haematologic toxicity grade 3 or 4, except grade 3 fatigue, transient arthralgia/myalgia, nausea/vomiting, diarrhoea, alopecia; treatment delay >2 weeks due to toxicity. The study maximum dose (SMD) was set to 30 mg kg^−1^ and maximum-tolerated dose (MTD) was defined as the dose level below any dose with >1 DLT. The rationale for the SMD was to reach up and above the trough level associated with efficacy in preclinical tumour xenograft models.

### PK assays

In the first three groups, serum PK samples were collected at 1, 4, 10, 24, and 72 h after the first infusion and then pre-dose and 60 min after start of subsequent infusions at weeks 1–3 and 6–11. Two additional PK samples were taken after the last dose week 7, at 4 and 48 h. For patients in dose groups 4 and 5, samples were taken after the first and last dose as described above, 2 h after the second dose, and weekly between dosing. TB-403 concentration was determined using an ELISA. The PK parameters were evaluated using a 2-compartment model (IV infusion, first order elimination, no lag time, weight 1/yhat^*^yhat) using the pharmacokinetic software WinNonLinPro v. 5.2 (Pharsight Corp., Mountain View, CA, USA). Actual sampling times were used for PK evaluation. Anti-TB-403 antibody titres were determined using a luminescence ELISA.

### Exploratory biomarkers

Levels of circulating leukocytes were analysed using flow cytometry. Living cells were gated based on the forward (reflecting size) and side (reflecting granularity) scatter distribution. In addition, out of the living cells, monocytes and granulocytes were defined based on the side scatter and expression of CD14 and lack of HLA-DR, respectively.

### Statistical analysis

Descriptive statistics were used for demographics, safety, and antitumour activity. Summary statistics on the PK parameters were presented using mean (arithmetic), median, CV%, max and min.

## Results

### Patient characteristics

Twenty-three patients were allocated to five dose groups. Demographics and baseline characteristics are listed in Table 1. Ten patients completed the visit at week 12 whereas 13 patients were withdrawn before week 12, all due to progressive disease or death. Six patients in dose groups 2, 3, and 5 showed stable disease at week 8 and continued on extended treatment with TB-403 (Table 2). One patient in dose group 2 went off study due to early progression after 2 weeks and was replaced, and one patient in dose group 5 developed tumour-related bile duct obstruction on day 2 and was also replaced.

### Safety

*DLT and SMD* No DLTs were observed and the MTD was not reached. A total of eight SAEs were reported in six patients. One occurred before the first dose and was not a treatment emergent adverse event (TEAE). Seven of the reported SAEs were considered unrelated to the study treatment. One was deemed possibly related and reported as an SUSAR (Serious Unexpected Suspected Adverse Reaction). This was a lung embolus in a patient with non-small cell lung cancer treated with a 10-mg kg^−1^ dose. The SAE occurred a few days after the patient had received the first dose of extended treatment and the treatment was stopped due to the event. Accordingly, this was not considered as a DLT. Three patients died during the study, two due to progressive disease (in dose group 2, 2.5 months after the first dose and in dose group 5, 1 month after the first dose) and one due to pneumonia and dysphagia (in dose group 1, 2.5 months after first dose). No patients were withdrawn due to AEs.

*Adverse events* In all, 232 TEAEs were reported by 22 of the 23 patients. The most commonly reported TEAEs (all grade 3–4 and >10% of the patient population for grade 1–2) are shown in Table 3. The most frequently occurring AEs were fatigue, constipation, pyrexia, dyspnoea, and nausea followed by cough.

In all, 101 AEs were considered as adverse drug reactions (ADRs). The highest incidence of ADRs was found in dose group 3 (10 mg kg^−1^) (41 ADRs, reported by three patients). Twenty-eight ADRs were reported by six subjects in the 30-mg kg^−1^ dose group.

Fatigue was the most commonly reported ADR, reported as 17 events by 9 patients (9 grade 1, 7 grade 2, and 1 grade 3). Nausea was reported as seven events of which six ADRs by six patients (5 grade 1 and 2 grade 2). Two of the ten events of pyrexia were considered as ADRs (grade 1) with one patient (10 mg kg^−1^) experiencing seven episodes of body temperature increase (grade 1). Six of these were considered as ADRs. One event was reported 2 weeks after the last dose and was considered as unrelated to TB-403. Three out of the ten events of dyspnoea were reported as ADRs (grades 1, 2, and 3). The grade 3 event was secondary to pneumonia and thus possibly remotely related. One of the ten events of constipation was reported as an ADR (grade 2). Hypersensitivity, urticaria, and rash were evaluated with special emphasis. Hypersensitivity as ADR was reported as two events for one patient (10 mg kg^−1^), both observed in connection with study drug administration (first and second doses). This patient had numerous body temperature rises following drug administration. One patient (10 mg kg^−1^) reported urticaria and itching with an onset 1 day after the first dosing. Both events were deemed related to TB-403. Two patients (20 mg kg^−1^ every third week) experienced isolated cases of rash (both grade 1) after TB-403 dosing (after first dose and second dose, respectively), both considered as related to treatment.

Overall, the severity as well as the causality of the AEs appeared to be unrelated to the dose of study medication – thus no trend across treatment groups were apparent, acknowledging the limited number of patients in each group. Six (6) events with a probable/certain relationship to study treatment were reported in four patients – one in dose group 1, four in dose group 3, and one in dose group 4. These included grade 1 peripheral neuropathy, grade 2 abdominal pain, grade 1 diarrhoea, grade 1 fatigue, grade 1 urticaria, and grade 1 fever. Adverse events encountered and possibly associated with angiogenesis inhibitors were hypertension grade 1, three events in one patient, upper GI haemorrhage grade 1, one event, proteinuria grade 2, one event, and pulmonary embolism grade 4, one event.

**Figure 2:**
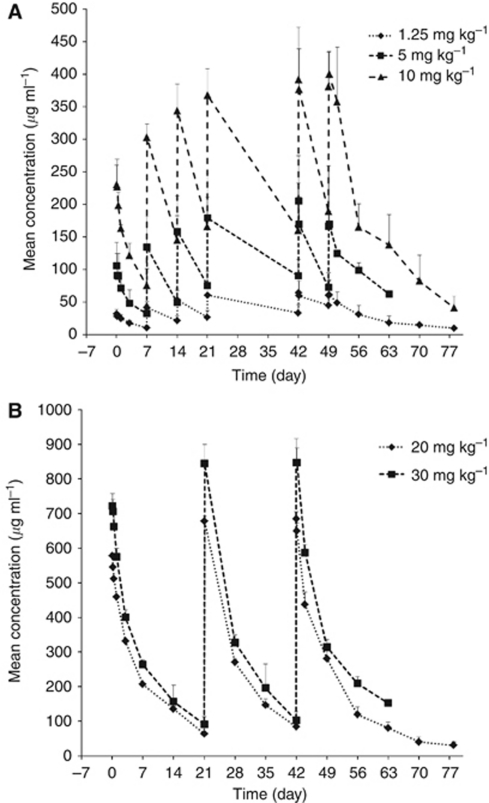
(**A**) TB-403 mean concentrations for dose groups A (1.25 mg kg^−1^), B (5 mg kg^−1^), and C (10 mg kg^−1^) *vs* time profiles using nominal time elapsed from dosing. (**B**) TB-403 mean concentrations for dose groups D (20 mg kg^−1^) and E (30 mg kg^−1^) *vs* time profiles using nominal time elapsed from dosing.

*Pharmacokinetics* The concentration data obtained allowed for a reliable estimation of the PK of TB-403 in all treatment groups, as analysed by fitting to a 2-compartment model for 18 out of the 23 patients. All patients had measurable serum TB-403 concentrations following dosing. The mean PK parameters are listed in Table 4 and the mean concentration–time profiles are illustrated in Figure 2.

Both maximum concentration (*C*_max_) and area under the concentration-time curve (AUC) values increased proportionally with dose. Following *C*_max_, concentrations generally declined in a biexponential pattern: a rapid distribution phase followed by a slow elimination phase. *C*_max_ and *T*_max_ were obtained directly from the serum concentration–time profile and time of maximum concentration was reached between the first and third blood sampling time (1, 4, or 10 h post dose).

The mean estimated half-life was found to be 9–14 days, independent of dose or body weight. The arithmetic and harmonic means for *t*_1/2_ based on all individuals (18) were 10 and 9.6 days, respectively. Hence, the terminal half-life was in the range of 1–2 weeks, which is similar to the half-life of several other therapeutic monoclonal antibodies.

Clearance and volume of distribution were found to be 4.0–5.7 ml kg^−1^ day^−1^ and 38–85 ml kg^−1^, respectively. This is on par with what is normally observed for therapeutic monoclonal antibodies. At TB-403 doses of 10 mg kg^−1^ weekly the trough level was higher (*C*_min_=189 *μ*g ml^−1^) than with doses of 30 mg kg^−1^ every third week (*C*_min_=102 *μ*g ml^−1^) (Table 4). Post-treatment anti-TB-403 binding antibodies in four patients did not appear to affect TB-403 exposure (data not shown).

*Exploratory biomarkers* Circulating leukocytes were analysed at baseline and throughout the study in 14 patients. In five of these, the numbers of monocytes were reduced (30–92% dose independent) after the first or second administration of TB-403. The reduction was more apparent after the second administration with a slower recovery compared with the first administration. This indicates that the effect would increase with repeated administrations but could not be confirmed as no samples were taken after subsequent TB-403 administrations.

*Antitumour activity* Objective tumour response was evaluated at week 8 and week 12. No objective responses according to RECIST 1.0 criteria were observed among the 23 patients. However, out of 20 patients assessed at week 8, 6 had stable disease and continued on extended treatment. Two of the patients, one with oesophageal squamous cell carcinoma and one with pancreatic adenocarcinoma, respectively, both treated with 5 mg kg^−1^ weekly, remained stable for 12 months. The patient with metastatic squamous cell carcinoma of the oesophagus initially received radiotherapy (60 Gy) and concomitant cisplatin for locally advanced disease, and had progressive disease after 5 months. He then received paclitaxel and capecitabine, but progressed after three cycles, before entering this study. Target lesions were enlarged lymph nodes in the mediastinum and retroperitoneum, and after 8 weeks of TB-403 a minor response of 25% was observed (best response s.d.). After 12 months on study, the patient progressed and went off study. The patient with pancreatic cancer had previously received concomitant radiotherapy and capecitabine for locally advanced disease and subsequently referred for surgery. However, a metastatic subcutaneously lesion was observed and resected with residual tumour cells, and radical resection of the primary tumour was therefore not attempted. The metastatic lesion then progressed and was target lesion during this study. The lesion remained stable for 12 months and then progressed. Two patients with prostate and salivary gland cancer, respectively, treated with 10 mg kg^−1^ every 3 weeks remained stable for 5 and 4 months, respectively. One of the patients with stable disease at week 8 died before week 12 due to disease progression. No trend in antitumour activity across dosing groups was evident.

## Discussion

TB-403 was generally well tolerated in this small population of heavily pretreated patients with advanced solid tumours. No AEs led to withdrawal from the study. The high number of TEAEs reflects the severely ill patient population. The majority of these events were either mild or moderate and more than half were considered unrelated to treatment. Three patients in three different groups (1.25, 5.0, and 30.0 mg kg^−1^) died during the study. The deaths were all considered related to disease progression and unrelated to TB-403. One patient on extended therapy was withdrawn after two doses in the extension part, due to an SAE. This was a patient with non-small cell lung cancer, who experienced a lung embolus possibly related to TB-403.

The most commonly observed TEAEs were fatigue, constipation, pyrexia, and dyspnoea followed by nausea and most frequently observed ADRs were fatigue, nausea, and body temperature increase. The MTD was not reached. However, there were generally more TEAEs and ADRs reported in dose group 3 (10 mg kg^−1^; q1w) than in dose group 5 (30 mg kg^−1^; q3w). However, overall exposure (AUC) is higher in dose group 5 compared with dose group 3. There was no obvious trend in vital signs, ECG, physical examination or laboratory values following TB-403 treatment. The study confirms our hypothesis that targeting PlGF is safe and different from anti-VEGF(R) therapies which are known to cause a number of side effects, including cardiotoxicity, heart failure, and hypertension (Eskens and Verweij, 2006; Force *et al*, 2007; Verheul and Pinedo, 2007; Eremina *et al*, 2008).

The clearance, volume of distribution, and terminal half-life of TB-403 were similar to other monoclonal antibodies. TB-403 serum concentrations that showed activity in preclinical models were attained. At weekly dosing with a dose level of 10 mg kg^−1^ or dosing q3w with 30 mg kg^−1^, trough serum concentrations exceeded 100 *μ*g ml^−1^ which is the level detected in preclinical tumour xenograft model experiments with optimal antitumour efficacy (data not shown). Four of the twenty-three patients were tested positive for antibodies against TB-403; however, no trend in relation to study treatment or across treatment groups was identified.

There is persuasive evidence that tumour-associated macrophages promote cancer initiation, malignant progression, and metastasis and the density of these cells in human solid tumours correlates with poor prognosis in a majority of cases (Mantovani *et al*, 1993, 2006; Bingle *et al*, 2002; Condeelis and Pollard, 2006). Placental growth factor has been shown to be important in the proangiogenic programming of such myeloid cells (Laurent *et al*, 2011). Furthermore, PlGF is known to be an important chemoattractant of VEGFR-1-positive bone marrow-derived myeloid cells (Kerber *et al*, 2008; Loges *et al*, 2009). Thus, one of the proposed mechanism of action of anti-PlGF antibodies is an effect on intratumoural macrophage recruitment. For example, reduced intratumoural macrophage recruitment accompanied by a normalising effect on the increased levels of circulating monocyte was shown in tumour model experiments (pancreatic and colon cancer) after anti-PlGF treatment, but not after anti-VEGFR-2 therapy (Fischer *et al*, 2007). Interestingly, in this study the levels of peripheral blood monocytes were analysed and found to be reduced in a group of patients after the first or second administration of TB-403, suggesting similar effects of TB-403 here as seen in the preclinical tumour models. Therefore, since recent studies have suggested that solid tumour angiogenesis and growth is highly dependent on non-tumour cells in the tumour microenvironment, particularly inflammatory cells of the myeloid lineage, which are known to be important sources of VEGF (Murdoch *et al*, 2008), an effect by TB-403 on macrophage recruitment and activation may provide therapeutic benefit in certain stages of tumour progression and should be further examined in future studies.

Although assessment of efficacy was not the primary objective of this study, TB-403 treatment revealed stable disease for 6 of the 23 evaluated patients at week 8. Two patients, one with oesophageal squamous cell carcinoma and the other with pancreatic adenocarcinoma, both treated with 5 mg kg^−1^ weekly, maintained stable disease for a total of 12 months. Two patients, one with prostate cancer and the other with salivary gland cancer and both treated with 10 mg kg^−1^ every 3 weeks, remained stable for a total of 5 and 4 months, respectively. No dose relationship was observed; however, in general the small group sizes did not allow any interpretation of the efficacy results.

Emerging data suggest that several pathophysiological mechanisms underlie the development of tumour resistance to therapy. Indeed, inhibitors of the VEGF pro-angiogenic signalling pathway have been recognised in the clinic to increase plasma levels of pro-angiogenic factors such as PlGF, which has been forwarded as one major determinant of drug-induced resistance to therapy (Bertollini *et al*, 2007). Thus, the safety profile of TB-403 described in this study and the anti-PlGF mode-of-action being distinct from that of anti-VEGF(R) inhibitors (Fischer *et al*, 2007), provide a good rationale for testing TB-403 in combination of with such drugs, and is currently being explored in clinical trials (ClinicalTrials.gov).

In summary, repeated intravenous administration of TB-403 was well tolerated in this fragile population of patients with advanced solid tumours and the experience with regard to safety profile of TB-403 appears to be in accordance with that expected from both healthy volunteers and preclinical data. In summary, TB-403 showed acceptable safety, tolerability, and appropriate PK profile, after repeated intravenous doses up to 30 mg kg^−1^, in patients with advanced solid tumours.

## Figures and Tables

**Table 1:** Patient demographics (*N*=23)

**Characteristics**	**Numbers**	**Age (years)**	**Previous chemotherapies**
*Gender*			
Male	11		
Female	12		
			
*Primary cancer*			
Bile duct cancer		51	3
Breast cancer		56	5
Breast cancer		67	2
Breast cancer		41	6
Colon cancer		71	4
Colon cancer		56	3
Hepatocellular carcinoma		62	1
Leiomyosarcoma		69	2
Medulloblastoma		40	8
Non-small cell lung carcinoma		63	5
Oesophageal carcinoma		49	2
Oesophageal carcinoma		53	7
Ovarian cancer		44	6
Ovarian cancer		55	6
Pancreatic carcinoma		62	2
Parotid adenocarcinoma		66	1
Penile carcinoma		63	5
Prostate cancer		65	3
Rectal cancer		64	5
Rectal cancer		64	5
Renal cancer		69	5
Thyroid cancer		62	0
Uterine cancer		54	6

**Table 2:** Time on study per dose group

**Dose group**	**TB-403 dose (mg kg^−1^ BW)**	**No. of patients**	**Patients completing week 8**	**Patients completing week 12**	**Median days on study**	**Patients on extension**
1	1.25	3	3	2	84	0
2	5.0	4^a^	3	3	225	2
3	10	3	3	3	85	1
4	20	3	1	0	41	0
5	30	10^a^	6	2	57	3

Abbreviation: BW=body weight.

a Two patients in dose groups 2 and 5, respectively, were withdrawn before day 29 and replaced by two new patients.

**Table 3:** Summary of TEAEs (grades 1–2 in >10% of the study population and all grades 3 and 4)

	**1.25 mg kg^−1^ *N*=3**		**5.0 mg kg^−1^ *N*=4**		**10 mg kg^−1^ *N*=3**		**20 mg kg^−1^ *N*=3**		**30 mg kg^−1^ *N*=10**		**Total *N*=23**
**Adverse event**	**Grades 1–2**	**Grades 3–4**	**Grades 1–2**	**Grades 3–4**	**Grades 1–2**	**Grades 3–4**	**Grades 1–2**	**Grades 3–4**	**Grades 1–2**	**Grades 3–4**	
Any	9	1	16	5	27	1	12	–	49	4	124
											
*GI toxicity*											
Constipation	–	–	1	–	2	–	2	–	5	–	10
Nausea	2	–	1	–	3	–	–	–	1	–	7
Vomiting	–	–	1	–	1	–	–	–	3	–	5
Dry mouth	–	–	–	–	1	–	–	–	2	–	3
Stomatitis	–	–	–	–	1	–	–	–	2	–	3
Diarrhoea	–	–	–	–	2	–	–	–	2	–	4
Abdominal pain	–	–	1	–	1	–	1	–	–	–	3
											
*General toxicity*											
Fatigue	1	–	3	–	10	–	2	–	5	2	23
Pyrexia	–	–	2	–	2	–	1	–	5	–	10
Pain	–	–	–	–	1	–	1	–	1	–	3
											
*Metabolic toxicity*											
Weight loss	2	–	–	–	1	–	–	–	2	–	5
Anorexia	–	–	1	–	2	–	–	–	3	–	6
Increased bilirubin	–	–	2	2	–	–	–	–	–	–	4
Increased ALP	1	–	–	2	–	–	–	–	–	–	3
Increased ALT	1	–	1	–	–	–	–		–	–	2
											
*Muscoloskeletal toxicity*											
Myalgia	–	–	1	–	–	–	–	–	4	–	5
Athralgia	–	–	–	–	–	–	1	–	2	–	3
Muscoloskeletal pain	–	–	–	–	–	–	–	–	2	1	3
											
*Respiratory toxicity*											
Dyspnoea	–	1	–	1	–	1	1	–	5	1	10
Cough	1	–	1	–	–	–	–	–	5	–	7
											
*Dermatological toxicity*											
Rash	1	–	1	–	–	–	3	–	–	–	5

Abbreviations: ALP=alkaline phosphatase; ALT=alanine transaminase; GI=gastrointestinal; TEAE=treatment emergent adverse event.

**Table 4:** Pharmacokinetic parameters

	**1.25 mg kg^−1^**	**5 mg kg^−1^**	**10 mg kg^−1^**	**20 mg kg^−1^**	**30 mg kg^−1^**
No. of patients	3	2	3	3	7
					
*T*_*max*_ *(hours)*					
Mean (arithmetic)	4.03	1.07^a^	2.12	1.12	2.8
Median	1.08	^−^	1.37	1.12	1.17
CV%	128	^−^	77.1	11.1	119
Max	10	^−^	4	1.25	9.88
Min	1	^−^	1	1	1
					
*C*_*max*_ *(μg ml*^−1^)					
Mean (arithmetic)	31.6	88.5	231	593	758
Median	27.8	88.5	235	616	752
CV%	21.1	4.28	15.4	9.82	16
Max	39.2	91.1	265	637	974
Min	27.6	85.8	194	527	630
					
AUC (day*μg ml^−1^)					
Mean (arithmetic)	319	882	1980	5020	6610
Median	327	882	1930	4960	6680
CV%	40	3.83	16.7	9.9	20.7
Max	442	906	2340	4960	8830
Min	188	858	1680	4550	4660
					
*V*_*ss*_ *(ml kg*^−1^)					
Mean (arithmetic)	53.5	100	71.2	47.3	59.2
Median	56.5	100	66.6	45.8	54.7
CV%	26.9	21	18.3	9.95	18.4
Max	66.1	115	85.9	52.6	71
Min	37.8	85.4	61	43.5	50.5
					
*CL (ml day^−1^ kg^−1^)*					
Mean (arithmetic)	4.44	5.68	5.14	4.01	4.72
Median	3.83	5.68	5.18	4.03	4.49
CV%	44.8	3.83	16.3	9.77	21.5
Max	6.66	5.83	5.95	4.39	6.44
Min	2.83	5.52	4.28	3.61	3.4
					
*t*_*1/2*_ *(days)*					
Mean (arithmetic)	9.36	13.4	10.6	8.99	9.88
Median	9.41	13.4	10.5	8.52	9.08
CV%	32.7	28	19.1	9.54	28.8
Mean (geometric)	9.01	13.1	10.5	8.96	9.55
Mean (harmonic)	8.67	12.8	10.4	8.94	9.25
Max	12.4	16	12.7	9.98	14.7
Min	6.28	10.7	8.69	8.47	6.89
					
*C*_*min*_ *(μg ml*^−1^)					
Mean (arithmetic)	45^b^	72^b^	189^b^	84^c^	102^c^
Median	35	83	164	81	108
CV%	81.6	30.9	30.3	7.97	26.1
Max	85.7	87.2	268	92	139
Min	14.5	46.7	135	79.6	69.1

Abbreviations: *T*_max_=time of maximum concentration after intravenous infusion; *C*_max_=maximum observed concentration after intravenous infusion; CV%=coefficient of variation; AUC=area under curve (the area under the concentration–time curve, from time zero to infinity); *V*_ss_=volume of distribution at steady state; CL=plasma clearance; *t*_1/2_=terminal half life

a One patient.

b Trough concentration is calculated as the mean of individual concentrations before dose 8 (*n*=3).

c Trough concentration is calculated as the mean of individual concentrations before dose 3 (*n*=3 for 20 mg kg^−1^ and *n*=8 for 30 mg kg^−1^).
